# Supplementary material for: “I’d rather wait and see what’s around the corner”: A multi-perspective qualitative study of treatment escalation planning in frailty
Source: PLoS One. 2023 Sep 21;18(9):e0291984. doi: 10.1371/journal.pone.0291984 (PMC10513333; doi:10.1371/journal.pone.0291984)
Supplement: Attachment — Submitted filename: Response to reviewers.doc [file pone.0291984.s001.doc]

Patient Experience Research Centre

School of Public Health

Imperial College London

London, W2 1PG, UK

21.07.2023

Sefonias Getachew, MPH, PhD
Academic Editor
PLOS ONE

Dear Sir,

Further to your email correspondence of 28.06.23 requesting revision of our manuscript to address points raised during the review process, we wish to re-submit the following original research article for consideration by PLOS One**:**

**“I’d rather wait and see what’s around the corner”: A multi-perspective qualitative study of treatment escalation planning in frailty.**

Our responses to the reviewer’s comments are detailed overleaf. Also overleaf, we address the additional journal requirements outlined in your mail.

We have uploaded a marked-up copy of our manuscript that highlights changes made to the original version. The file is labelled 'Revised Manuscript with Track Changes'.

We have uploaded an unmarked version of our revised paper without tracked changes. The file is labelled 'Manuscript'.

Please address all correspondence concerning this manuscript to [a.lound@imperial.ac.uk](mailto:a.lound@imperial.ac.uk)

Thank you for your consideration of this manuscript.

Sincerely,

Adam Lound

Research Physiotherapist

Patient Experience Research Centre

Imperial College London

Response to reviewer comments:

| Reviewer | Comment | Response |
| --- | --- | --- |
| Reviewer #1: | This paper presents an important contribution in understanding treatment escalation among persons with frailty. However, as written the methods are not technically sound. The authors need to explain how methods of phenomenology are applied within the framework analysis as the underlying methodologies of phenomenology and framework analysis are incongruent. Phenomenology typically uses unstructured interviews to develop a deep understanding, or essence, of a phenomenon and requires the researcher to carefully bracket personal biases. In contrast, framework analysis is driven by a priori ideas and pre-determined questions. As a result of this lack of clarity on methods, the results are not presented in a manner that is congruent with either method. The finding presented are rich and meaningful, and I encourage the authors to choose one qualitative method and re-analyze the data. | We thank the reviewer for highlighting this oversight in our written methods. We wish to clarify our approach.  We acknowledge that we did not undertake a phenomenological approach in this study and did not provide enough detail about how we utilised the Framework Method.  We incorrectly used the term phenomenology to describe that our analysis was guided by our research aim to explore *participant perspectives* of treatment escalation in the context of frailty. We have removed the reference to phenomenology and have added a more detailed summary of the Framework Method and how we used it in this study, in particular the development of a matrix based on key areas from the topic guide.  The reworded section below provides clarity on our analysis approach. We are confident that we have undertaken a thoughtful and rigorous analysis and, in our opinion, re-analysing the data would not add any additional value to this paper.  **The original wording was as follows:**  Transcripts were uploaded and managed using NVivo (QSR International (UK) Ltd) and analysed using the Framework Method (18). We approached our analysis though a phenomenological perspective, aiming to gain an in-depth understanding of individuals’ lived experiences and to explore how they constructed meaning in relation to frailty and planning for treatment escalation (19). Familiarisation and coding were undertaken by two researchers (AL and KJ) with 19 of the 25 transcripts double coded. Codes were then indexed and charted (AL) within three major headings: frailty, acute crisis and prospective planning. One researcher (AL) created a framework matrix for each stakeholder group and a separate framework for the focus groups. Three research team members (AL, KJ, JB) met regularly to develop the analytic framework and clarify themes and interpretation of the findings. Findings were presented to the wider research team and public advisors for review.  **The updated wording is as follows:**  Transcripts were uploaded and managed using NVivo (QSR International (UK) Ltd) and analysed using the Framework Method. The Framework Method is a systematic approach to qualitative analysis which includes the use of a matrix to organise and summarise data. Familiarisation and coding were undertaken by two researchers (AL and KJ) with 19 of the 25 transcripts double coded. Codes were then indexed and charted (AL) into a matrix based on the key areas explored in the topic guides: frailty, acute crisis and prospective planning. One researcher (AL) created a matrix for each stakeholder group and a separate matrix for the focus groups. Three research team members (AL, KJ, JB) met regularly to iteratively develop the analytic framework and clarify themes and interpretation of the findings. Findings were presented to the wider research team and public advisors for review.  1. Gale NK, Heath G, Cameron E, Rashid S, Redwood S. Using the framework method for the analysis of qualitative data in multi-disciplinary health research. BMC Med Res Methodol. 2013 Dec;13(1):117. |
| Reviewer #2: | The manuscript is technically sound. Under Abstract section: please try to further explain the Methods part and the Conclusion: Can you clearly state your relevant findings and recommendations? Can you remove the bullet points you used to present the themes? Please write them in sentence form. | Thank you for this feedback on how to improve our abstract. We have added detail to the Methods section and rewritten the conclusion to better summarise our findings and recommendations.  We have removed the bullet points from the findings section.  **The original abstract was as follows:**  **Background:** People living with frailty risk adverse outcomes following even minor illnesses. Admission to hospital or the intensive care unit is associated with potentially burdensome interventions and poor outcomes. Decision-making during an emergency is fraught with complexity and potential for conflict between patients, carers and clinicians. Advance care planning is a process of shared decision-making which aims to ensure patients are treated in line with their wishes. However, planning for future care is challenging and those living with frailty are rarely given the opportunity to discuss their preferences.  **Aim:** The aim of the ProsPECT (Prospective Planning for Escalation of Care and Treatment) study was to explore perspectives on planning for treatment escalation in the context of frailty. We spoke to people living with frailty, their carers and clinicians across primary and secondary care.  **Setting:** Two large NHS Trusts based in Northwest London.  **Participants:** 44 participants: 9 patients, 11 carers and 24 clinicians  **Methods:** In-depth interviews and focus groups thematically analysed using a framework approach.  **Findings:** We identified four main themes: 1) Frailty is absent from treatment escalation discussions, 2) Planning for an uncertain future, 3) Escalation in an acute crisis is *‘the path of least resistance’,* and 4) Approaches to facilitating treatment escalation planning in frailty.  **Conclusions:** This qualitative study explored different perspectives on frailty and identified barriers to planning for escalation related to negative perceptions of the condition and uncertainty. It provides insights into the challenges of decision-making in an emergency and discuss potential ways to better facilitate future planning.  **The new abstract is as follows:**  **Background:** People living with frailty risk adverse outcomes following even minor illnesses. Admission to hospital or the intensive care unit is associated with potentially burdensome interventions and poor outcomes. Decision-making during an emergency is fraught with complexity and potential for conflict between patients, carers and clinicians. Advance care planning is a process of shared decision-making which aims to ensure patients are treated in line with their wishes. However, planning for future care is challenging and those living with frailty are rarely given the opportunity to discuss their preferences.  **Aim:** The aim of the ProsPECT (Prospective Planning for Escalation of Care and Treatment) study was to explore perspectives on planning for treatment escalation in the context of frailty. We spoke to people living with frailty, their carers and clinicians across primary and secondary care.  **Setting:** Two large NHS Trusts based in Northwest London.  **Participants:** 44 participants: 9 patients, 11 carers and 24 clinicians.  **Methods:** In-depth online or telephone interviews and online focus groups. The topic guide explored frailty, acute decision-making and planning for the future. Data were thematically analysed using the Framework Method. Preliminary findings were presented to a sample of study participants for feedback in two online workshops.  **Findings:** We identified four main themes: Frailty is absent from treatment escalation discussions, planning for an uncertain future, escalation in an acute crisis is *‘the path of least resistance’,* and approaches to facilitating treatment escalation planning in frailty.  **Conclusions:** Barriers to treatment escalation planning include a lack of shared understanding of frailty and uncertainty about the future. Emergency decision-making is focussed on survival or risk aversion and patient preferences are rarely considered. To improve planning discussions, we recommend frailty training for non-specialist clinicians, multi-disciplinary support, collaborative working between patients, carers and clinicians as well as broader public engagement. |

Response to additional journal requirements:

| Number | Comment | Response |
| --- | --- | --- |
| 1 | Please ensure that your manuscript meets PLOS ONE's style requirements, including those for file naming. | We have reviewed our document and made sure our manuscript aligns with PLOS ONE’s style requirements.  During this review we also noted and changed some minor issues in our text such as incorrect spacing or capitalisation. These changes can be clearly seen in the track changes document. |
| 2 | We note that the grant information you provided in the ‘Funding Information’ and ‘Financial Disclosure’ sections do not match. | Thank you - we have rewritten the statement and would like this to be used for both our Financial Disclosure and Funding Information:  This project was funded by two grants, HW received funding from the NIHR Imperial Biomedical Research Centre (NIHR203323) <https://imperialbrc.nihr.ac.uk/>. SJB received funding from the Imperial College Healthcare Trust Research Capability Fund (WSAA_P83429) <https://www.imperial.nhs.uk/>. The funders had no role in study design, data collection and analysis, decision to publish, or preparation of the manuscript. HW is an NIHR Senior Investigator and acknowledges support from the NIHR ARC Northwest London and the NIHR School of Public Health Research. BP receives general support from the UKRI CDT in AI for Healthcare (EP/S023283/1) [http://ai4health.io](http://ai4health.io/). |
| 3 | In your Data Availability statement, you have not specified where the minimal data set underlying the results described in your manuscript can be found. | We have updated our data availability statement as follows:  This is a qualitative study and the full study data cannot be made publicly available due to the sensitive nature of this data, the agreements and procedures governing usage and sharing of the collected dataset established by the research institution (Imperial College London) and Research Ethics Board (21/LO/0125). However, we welcome approaches to the Patient Experience Research Centre from bona fide researchers who may wish to explore the data further in collaboration with our team. Interested parties should provide a data access request to [patientexperience@imperial.ac.uk](mailto:patientexperience@imperial.ac.uk) and following approval of the request, establish a data-sharing agreement between the investigators and the Study PIs. |
| 4 | We note that Figure 1 in your submission contain copyrighted images. | This figure has now been recreated using icons from <https://healthicons.org/> Which clearly states the following: *Free for use in your next commercial or personal project. Editing is ok. Republishing is ok. No need to give credit.* The new figure is as follows: \|  \| **1: Frailty is absent from treatment escalation discussions** *Patients and Carers: ‘We don’t accept that’* *Clinicians: Lacking confidence and ‘skirting around the issue’*; **Prospective Planning for Escalation of Care and Treatment in Frailty** \|  \| **2: Planning for an uncertain future** *Patient and carer uncertainty: ‘I’d rather wait and see what’s around the corner’* *Clinician uncertainty: ‘A fantastically complex scenario’* *Clashing perspectives: Potential for conflict when making planning for treatment escalation*; \|  \| **3: Treatment escalation in an acute crisis is *‘the path of least resistance’*** *Focus on survival* *Risk Aversion leads clinicians to ‘err on the side of doing everything’*; \|  \| **4: Approaches to facilitating treatment escalation planning in frailty** *Content: Fostering a shared understanding* *Context: Timing, trust and support* |
