# Supplementary material for: “I’d rather wait and see what’s around the corner”: A multi-perspective qualitative study of treatment escalation planning in frailty
Source: PLoS One. 2023 Sep 21;18(9):e0291984. doi: 10.1371/journal.pone.0291984 (PMC10513333; doi:10.1371/journal.pone.0291984)
Supplement: Attachment — Submitted filename: Response to reviewers.doc [file pone.0291984.s002.doc]

Patient Experience Research Centre

School of Public Health

Imperial College London

London, W2 1PG, UK

25.08.2023

Sefonias Getachew, MPH, PhD
Academic Editor
PLOS ONE

Dear Sir,

Further to your email correspondence of 22.08.23 requesting revision of our manuscript to address PLOS One publishing guidance, we wish to re-submit the following original research article for consideration**:**

**“I’d rather wait and see what’s around the corner”: A multi-perspective qualitative study of treatment escalation planning in frailty.**

Our responses to the comments are detailed overleaf.

We have uploaded a marked-up copy of our manuscript that highlights changes made to the original version. The file is labelled 'Revised Manuscript with Track Changes'.

We have uploaded an unmarked version of our revised paper without tracked changes. The file is labelled 'Manuscript'.

Please address all correspondence concerning this manuscript to [a.lound@imperial.ac.uk](mailto:a.lound@imperial.ac.uk)

Thank you for your consideration of this manuscript.

Sincerely,

Adam Lound

Research Physiotherapist

Patient Experience Research Centre

Imperial College London

| Section | Editors Comments | Response |
| --- | --- | --- |
| Abstract | Please revise some of the sections in your manuscripts. You are advised to amend again the abstract section and structure it as the introduction, method, result, and concussion sections. Keep the keywords as indicated at the end. | We have now revised the abstract as suggested. |
| Main document | In the main document, again the heading used as finding is to be changed to ‘Result’. | We have now changed the heading ‘Findings’ to Results’ as suggested. |
| Discussion | It is good to remove also the subheadings stated as main findings, and comparison to other literature, and others in the discussion section. | We have now removed all subheadings from the discussion section as suggested. |
| Figure | Remove also the Figure 1 included in the document and submit it as a separate file in the system based on instructions for Figure documents. However, you have to state the caption and place where the figure to be kept | We have removed Figure 1 from the manuscript, leaving the Figure caption immediately after when the figure is first cited in the text as per PLOS One guidance.  We have uploaded the figure and converted it using the PACE tool as suggested. This has been uploaded as a tif file marked as Fig 1. |
